# Human Wild-Type α-Synuclein Drives Progressive Tau Pathology in a Disease-Relevant Mouse Model of Synucleinopathy

**DOI:** 10.21203/rs.3.rs-8166445/v1

**Published:** 2025-12-03

**Authors:** Sudipta Senapati, Alessia Sciortino, Madison Samples, Nikita Shchankin, Yingxin Zhao, Mauro Montalbano, Rakez Kayed

**Affiliations:** University of Texas Medical Branch; University of Texas Medical Branch; University of Texas Medical Branch; University of Texas Medical Branch; University of Texas Medical Branch; University of Texas Medical Branch; University of Texas Medical Branch

**Keywords:** Tau pathology, α-Synuclein, Parkinson’s disease, Synucleinopathy, Co-pathology, Neurodegeneration, hyperphosphorylation, insoluble tau, neurofibrillary tangles

## Abstract

Tau pathology, characterized by the aberrant aggregation and accumulation of tau in neurons and glial cells, plays a critical role in the onset and progression of multiple neurodegenerative diseases. Although synucleinopathies such as Parkinson’s disease (PD) and dementia with Lewy bodies (DLB) are primarily defined by intracytoplasmic inclusions of α-Synuclein (α-Syn), they frequently exhibit substantial tau co-pathology. Emerging genetic and pathological evidence suggests a mechanistic interplay between α-Syn and tau that amplifies their aggregation and accelerates neurodegeneration. In this study, we systematically investigated the temporal progression of tau pathology in a transgenic PD mouse model that overexpresses human wild-type (WT) α-Syn (hSyn mice). Prior work has shown that this model develops α-Syn pathology across key brain regions, including the substantia nigra, cortex, hippocampus, and striatum, accompanied by microglial activation and synaptic dysfunction. Using a combination of biochemical, biophysical, and immunological approaches, we demonstrate a progressive accumulation of hyperphosphorylated tau, as well as soluble and insoluble tau aggregates, in the brains of hSyn mice. Electron microscopy of insoluble fractions reveals abundant fibrillar structures, while mass spectrometry confirms that these fibrils consist of both α-Syn and tau. Notably, these pathologies are absent in WT littermates, suggesting that tau aggregation arises as a consequence of α-Syn overexpression rather than normal aging. Collectively, our findings establish a mechanistic link between α-Syn and tau aggregation, identifying tau as an active contributor to α-Syn–driven neurodegeneration. This study provides direct experimental evidence that tau co-pathology contributes to disease progression in synucleinopathies, underscoring the therapeutic potential of targeting pathological tau to mitigate neurodegeneration in PD, DLB, and related disorders.

## Introduction

Tau pathology, characterized by the abnormal aggregation and accumulation of tau protein in neurons and glial cells, plays a central role in the onset and progression of several neurodegenerative diseases [1]. These include classical tauopathies such as Alzheimer’s disease (AD), frontotemporal dementia (FTD), progressive supranuclear palsy (PSP), and corticobasal degeneration (CBD) [1–3]. In healthy neurons, tau is a microtubule-associated protein essential for maintaining neuronal structure, modulating neurite outgrowth, regulating axonal transport, and supporting synaptic integrity [4]. Its function is tightly regulated through a series of post-translational modifications (PTMs), including phosphorylation, acetylation, methylation, nitration, ubiquitination, sumoylation, and truncation [5, 6]. Under pathological conditions, dysregulation of these PTMs alters tau’s charge, solubility, and conformation, promoting its detachment from microtubules and subsequent aggregation into soluble aggregates and finally into insoluble neurofibrillary tangles (NFTs) [6–8]. Among these PTMs, hyperphosphorylation is the most extensively studied and is strongly implicated in tau misfolding and aggregation [9]. Hyperphosphorylated tau assembles into toxic aggregates and NFTs, which disrupt cellular homeostasis, impair synaptic function, trigger neuroinflammation, and contribute to progressive neuronal loss and brain atrophy [1, 10].

Although tau pathology is most prominently associated with classical tauopathies, growing evidence suggests that it also contributes to synucleinopathies, including Parkinson’s disease (PD) and dementia with Lewy bodies (DLB) [1, 11, 12]. These disorders are classically defined by intracytoplasmic inclusions of α-Synuclein (α-Syn) within neurons and glia; however, tau aggregates are frequently detected in the same affected brain regions [11, 13]. Our previous work demonstrated that soluble aggregates of α-Syn and tau co-occur in PD and DLB human brains, highlighting the contribution of tau in synucleinopathies [14]. In these disorders, hyperphosphorylated tau frequently co-localizes with α-Syn inclusions or coexists in its proximity [15–17]. Subsequent studies have confirmed that α-Syn and tau interact, and cross-seed each other’s aggregation *in vitro* and *in vivo*, amplifying their mutual toxicity and contributing to a more complex and severe disease phenotype [14, 18–20].

Preclinical animal studies have provided compelling evidence that tau pathology is a critical component of α-Syn-driven neurodegeneration [21–24]. Transgenic mice overexpressing mutant forms of α-Syn, such as A30P, A53T, and E46K, develop hyperphosphorylated tau concurrently with α-Syn inclusions [25–27]. Synaptic recordings from hippocampal slices and cultured neurons from A53T mice show tau-dependent postsynaptic dysfunction [28], while tau alterations in these models are associated with elevated activity of kinases such as GSK3β and CDK5, and the formation of insoluble tau aggregates that co-localize with α-Syn inclusions [26, 29]. Notably, tau appears essential for the synaptic and memory deficits observed in A53T mice [30].

Despite these advances, the temporal progression and regional vulnerability of tau pathology in models overexpressing human WT α-Syn remain poorly understood. This is particularly important because mutations in the α-Syn gene (*SNCA*) account for less than 1% of all PD cases, whereas the vast majority (~ 85–90%) are sporadic, arising from a complex interplay of genetic predisposition and environmental factors that drive age-related dysregulation of WT α-Syn expression, clearance, and aggregation [31–33]. In contrast to mutant α-Syn models that exhibit early-onset and aggressive pathology, WT α-Syn models better replicate the progressive and age-dependent nature of human synucleinopathies. Hence, they offer a more physiologically relevant framework for investigating the emergence and progression of tau pathology and the mechanistic interplay between α-Syn and tau in disease-relevant contexts.

The co-occurrence of tau and α-Syn pathologies is associated with more severe clinical symptoms, greater diagnostic complexity, increased disease heterogeneity, and reduced treatment efficacy [34, 35]. Understanding tau’s role in this context is therefore essential for elucidating disease mechanisms and identifying effective therapeutic targets. In this work, we studied the temporal evolution of tau pathology in a transgenic α-Syn mouse model of PD (mThy1-hSNCA, abbreviated as hSyn), which overexpresses human WT α-Syn [36, 37]. This model is characterized by the development of α-Syn pathology in several brain regions, including the striatum, midbrain, hippocampus, and cortex [37, 38], along with motor and olfactory dysfunction [38–40], and microgliosis [36]. Using a combination of biochemical assays, immunohistochemical analyses, and structural approaches, we systematically characterized the temporal progression of tau pathology in hSyn mice. Our findings demonstrate an age-dependent accumulation of hyperphosphorylated and aggregated tau, coinciding with increasing α-synuclein pathology. This progressive tauopathy, absent in age-matched WT controls, provides a mechanistic link between α-Syn and tau aggregation, reinforcing its role as a key contributor to synucleinopathy-related neurodegeneration.

## Materials and methods

### Animals

The mThy1-hSNCA (JAX #017682) and WT C57BL/6NJ mice were obtained from the Jackson Laboratory and housed at the University of Texas Medical Branch (UTMB) animal facility. Genotyping was performed on all offspring via PCR analysis using DNA extracted from ear-punch samples. All experimental procedures adhered strictly to ARRIVE guidelines and National Institutes of Health (NIH) standards for the care and use of laboratory animals. Protocols were approved by the Institutional Animal Care and Use Committee (IACUC) to ensure ethical and humane treatment throughout the research. Only male mice were used in this study. Age groupings were designated as follows: mice aged 3–5 months were classified as the 5-month group, those aged 8–12 months as the 10-month group, and mice aged 18–22 months as the 20-month group. Mice were euthanized and perfused for brain collection. The left-brain hemisphere was snap-frozen at − 80°C for biochemical analysis, while the right hemisphere was embedded in OCT, sectioned, and used for immunofluorescence and immunohistochemistry studies.

### Brain homogenate preparation

Brain homogenates were prepared following previous protocols [41, 42]. Briefly, hemi-brains were homogenized using a TissueLyser LT (QIAGEN) at 50 pulses/s in phosphate-buffered saline (PBS) containing a protease/phosphatase inhibitor cocktail (Roche, Germany) at a 1:3 brain-to-PBS dilution ratio (w/v). The homogenates were then centrifuged at 10,000 × g for 10 minutes at 4°C to separate the PBS-soluble proteins from the pellet. Supernatants were collected and stored at − 80°C for later analysis.

### Preparation of sarkosyl-soluble and insoluble fractions

Sarkosyl-soluble and -insoluble fractions were prepared as described previously with minor modifications [43]. To extract sarkosyl-soluble and -insoluble proteins, PBS-pellets were resuspended in PHF extraction buffer (10 mM Tris-HCl, 10% (w/v) sucrose, 0.85 M NaCl, 1 mM EDTA, pH 7.4) at a 1:5 (w/v) ratio and homogenized using a TissueLyser. The homogenates were centrifuged at 10,000 × g for 10 minutes at 4°C for 3 times, after which sarkosyl was added to the supernatant to a final concentration of 1%. This solution was stirred at room temperature for 1 hour and then subjected to ultracentrifugation at 300,000 × g for 1 hour at 4°C, separating the sarkosyl-soluble fraction (in the supernatant) from the sarkosyl-insoluble fraction, which was collected by resuspending the resulting pellet in 1X PBS.

### Immunofluorescence assay of brain tissues

Immunofluorescence assays were conducted on frozen sections (12 μm thick) of mouse brains. The sections were first fixed in chilled methanol, then blocked in a buffer containing 5% BSA, 5% normal goat serum, and 0.25% Triton X-100 in 1X PBS (PBST) for 1 hour at room temperature. Following blocking, the sections were incubated with a Mouse-on-Mouse (MoM) blocking solution (one drop of MoM solution in 4 ml PBST, Vector Laboratories; MKB-2213) for 1 hour at room temperature. Next, sections were incubated overnight at 4°C with primary antibodies diluted in 5% BSA in PBST, including mouse anti-phospho-tau clone AT8 (1:500, Invitrogen; MN1020), mouse anti-phospho-tau clone AT100 (1:500, Invitrogen; MN1060), and chicken polyclonal anti-tau (1:500, Abcam; ab75714). After three 10-minute washes in 1X PBS, sections were incubated with Alexa-conjugated secondary antibodies (1:700, Invitrogen) diluted in 5% BSA in PBST for 1 hour at room temperature. Finally, the slides were washed, mounted with Prolong Gold antifade reagent with DAPI (Invitrogen, P36935), and prepared for imaging. Images were captured on a Keyence BZ-800 microscope with a 40× objective and analyzed using Fiji ImageJ software (NIH).

### Immunocytochemistry assay

For the immunocytochemistry assay, brain sections (12 μm thick) were fixed in chilled methanol, washed in PBS, and blocked for 1 hour at room temperature in a blocking buffer containing PBST with 5% normal goat serum and 5% BSA. After blocking, sections were incubated for 1 hour at room temperature with MoM blocking solution. Sections were then incubated overnight at 4°C with primary antibodies mouse anti-phospho-tau clone AT8 (1:300, Invitrogen; MN1020) and rabbit-phospho-tau-Thr231 (pThr231, 1:250, Invitrogen; 701056) diluted in 5% BSA in PBST. The following day, sections were washed three times in PBS for 10 minutes each and incubated for 1 hour with biotinylated horse anti-mouse or anti-rabbit IgG (1:5000, Vector Laboratories; PK-4001, PK-4002). After another series of three 10-minute PBS washes, the sections were visualized using an ABC reagent kit (Vector Laboratories) following the manufacturer’s protocol. Sections were washed in PBS and stained with DAB. Finally, sections were counterstained with hematoxylin (Vector Laboratories; H-3401) for nuclear staining and mounted for imaging. Images were acquired using a Keyence BZ-800 microscope with a 40× objective and subsequently analyzed with Fiji ImageJ software (NIH).

### Western blot analysis

Equal amounts of PBS-soluble fractions from mouse brain homogenates were loaded onto precast NuPAGE 4–12% Bis-Tris gels (Invitrogen) for SDS-PAGE, followed by transfer to nitrocellulose membranes. Membranes were blocked for 1 hour at room temperature using Odyssey Blocking Buffer (LI-COR; 927–60001) and then incubated overnight at 4°C with primary antibodies diluted in Odyssey Antibody Diluent (LI-COR; 927–65001). Secondary IRDye antibodies (LI-COR; 926–32210, 926–32211) were applied at a 1:10,000 dilution for 1 hour at room temperature. Primary antibodies used in this analysis included mouse anti-phospho-tau clone AT8 (1:500, Invitrogen; MN1020), mouse anti-phospho-tau clone AT100 (1:500, Invitrogen; MN1060), rabbit anti-phospho-tau (Thr231) pThr231 (1:250, Invitrogen; 701056), mouse anti-Tau-13 (1:1000, BioLegend; MMS-520R), rabbit polyclonal anti-tau (1:1000, Abcam; ab64193), mouse anti-Tau-5 (1:1000, Kanaan), and rabbit anti-β-actin (1:5000, Abcam; ab8227). Images were acquired using a LI-COR Odyssey imager, and densitometric analysis was performed with ImageJ software (NIH).

### Filter trap assay

The filter trap assay was conducted using a Bio-Dot SF microfiltration apparatus (Bio-Rad) following previous protocols [44, 45]. Briefly, a nitrocellulose membrane was cut to fit the slot blot chamber and pre-soaked in 1X Tris-buffered saline with 0.01% of Tween (TBS-T) for 5 minutes. Samples of PBS-soluble, sarkosyl-soluble, and sarkosyl-insoluble proteins, alongside recombinant tau fibrils and PBS as controls, were loaded in duplicate into the wells of the slot blot apparatus and filtered via vacuum. The membrane was then blocked with Odyssey blocking buffer (LI-COR; 927–60001) and incubated overnight at 4°C with primary antibodies diluted in Odyssey antibody diluent. The primary antibodies used were rabbit polyclonal anti-tau (1:1000), mouse anti-Tau-5 (1:5000; Kannan), and rabbit polyclonal anti-α-Syn (1:1000; Proteintech 10842–1-AP). On the following day, the membrane was washed three times with TBS-T and then incubated with secondary IRDye antibodies at a 1:10,000 dilution for 1 hour at room temperature. After a final series of three washes in TBS-T, images were acquired using the LI-COR Odyssey imager.

### Mass Spectrometry

Mass spectrometry was performed following previous protocols [43, 46]. Desalted peptides were reconstituted in 20 μL of 4% acetonitrile (ACN) with 0.1% formic acid. Peptides (10 μL) were loaded onto a trap column (Acclaim^®^ PepMap 100, 75 μm × 2 cm, C18, 3 μm) and separated on an analytical column (75 μm × 25 cm, C18, 2 μm) using an Easy-nLC 1000 UHPLC system (Thermo Scientific) at 300 nL/min. A 2-hour linear gradient (2–35% ACN in 0.1% formic acid) was applied. Data were acquired on a Q Exactive Orbitrap mass spectrometer (Thermo Scientific) in positive ion mode using Xcalibur 2.3. Full MS scans (m/z 350–1600) were collected at 70,000 resolution (AGC 1e6, max IT 20 ms), followed by HCD MS/MS of the top 15 ions (resolution 17,500, AGC 1e5, max IT 200 ms, NCE 28). Dynamic exclusion was set to 30 s. Raw data were analyzed with MaxQuant (v1.5.2.8) [47] using the Andromeda search engine against the Human SwissProt database (20,193 entries), plus common contaminants and reverse sequences. Trypsin specificity was used (max 2 missed cleavages); carbamidomethylation of cysteine was fixed, and methionine oxidation was variable. Precursor and fragment mass tolerances were 10 ppm and 0.5 Da, respectively. Label-free quantification was performed using MaxQuant’s LFQ algorithm with “match between runs” enabled (RT window: 0.7 min) [48]. FDR was set to 1% at peptide and protein levels; minimum peptide length was six amino acids. Reverse hits, contaminants, and proteins identified only by modified peptides were excluded. Data was processed in Perseus platform [49] to analyze the Maxquant output and perform statistical analysis. Proteins with fewer than three valid values in at least one experimental group were excluded. Remaining missing values were imputed from a normal distribution (width = 0.3×SD, downshift = 1.8×SD). Intensity of each α-Syn peptide was normalized with the proteotypic peptide EGVLYBGSK [50], while IGSLDNITHVPGGGNK was used for tau [51]. Differential expression of tau peptides was assessed using a multiple-sample ANOVA with permutation-based FDR correction.

### Electron Microscopy

Carbon-coated Formvar copper grids (200 mesh) were glow-discharged for 30 seconds using the PELCO easiGlow^™^ system. A 5 μL aliquot of the samples was applied to glow-discharged grids and allowed to adsorb for 3 minutes. Excess liquid was removed by gently blotting the edge of the grid with filter paper. The grids were then rinsed with 30 μL droplets of distilled water, followed by a brief wash in molecular grade water. For staining, the grids were placed on a fresh 30 μL droplet of 2% uranyl acetate for 30 s. The remaining stain was wicked off, and the grids were air-dried. Imaging was performed using a JEM-1400 transmission electron microscope (JEOL, Japan) operated at 80 kV, and micrographs were captured using an Orius SC2001 digital camera (Gatan, Pleasanton, CA).

### Statistical analysis

GraphPad Prism 9.0 software was used to generate graphs and perform statistical analyses. Data are presented as means ± SEM. Depending on the experimental design, statistical comparisons were made using either an unpaired, two-tailed Student’s t-test or one-way analysis of variance (ANOVA), followed by Tukey’s post-hoc test for multiple comparisons. Statistical significance was defined as p < 0.05. Specific details for each statistical test applied in individual experiments are provided in the corresponding figure legends.

## Results

The hSyn mouse (C57BL/6N-Tg (Thy-1-SNCA)15Mjff/J, JAX #017682) overexpresses human WT α-Syn in a neuron-specific manner and exhibits progressive pathological and behavioral alterations with age [36–40]. α-Syn pathology is detected in key brain regions, including the cortex, hippocampus, and substantia nigra, by 6 months [38], accompanied by reductions in dopamine and serotonin levels by 12 months [37]. Microglial activation becomes evident in the striatum by 10 months of age [36]. Behaviorally, hSyn mice exhibit significant reductions in total locomotor activity and ambulation after 8 months [37], along with marked impairments in motor coordination and olfactory function [38–40]. By 18 months, cortical cerebral blood flow (CBF) decreases by approximately 36.6%, with an overall reduction of about 28% in whole-brain CBF [40].

In line with these findings, we observed that α-Syn protein levels were significantly higher in hSyn mice compared to WT littermates as early as 5 months of age, with levels continuing to increase over time (Supplementary **Fig. S1A–C**), indicating an age-dependent accumulation of α-Syn pathology in hSyn mice.

### Age-dependent accumulation of hyperphosphorylated tau and NFTs in hSyn mice

Multiple studies have reported tau pathology in post-mortem brains of individuals with synucleinopathies [11, 13, 14, 35, 52, 53] and animal models of PD [23, 27, 28, 30]. Previous findings from our lab have also demonstrated that tau and α-Syn co-exist within the same pathological aggregates in PD and DLB cases [14].

**Figure 1:**
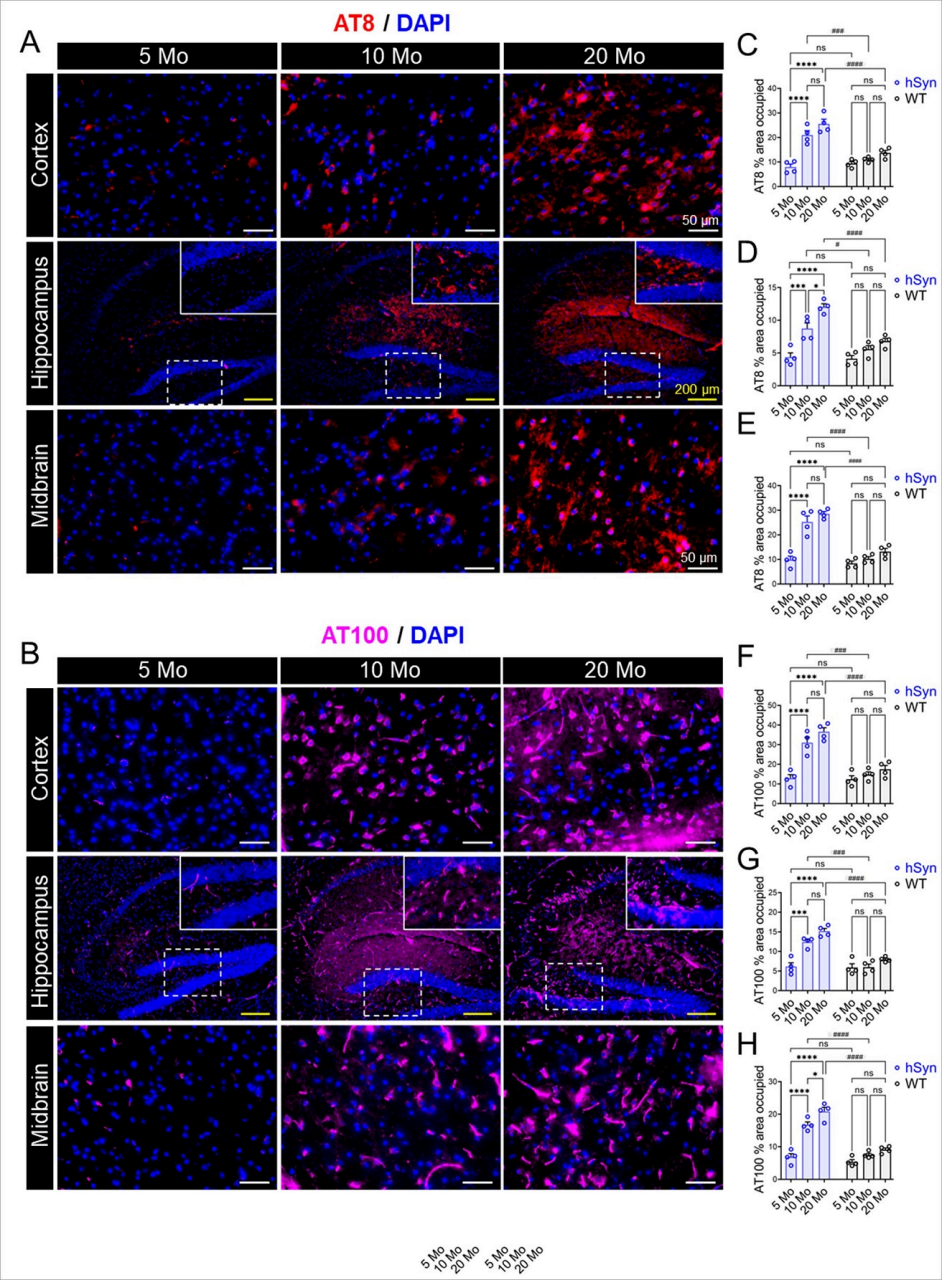
Age-dependent accumulation of hyperphosphorylated tau in the hSyn transgenic mice. (**A**, **B**) Representative immunofluorescence images showing a progressive, age-dependent increase in hyperphosphorylated tau (AT8 and AT100 immunoreactivities) in the cortex, hippocampus, and midbrain of 5-, 10-, and 20-month-old hSyn mice. (**C-E** & **F-H**) Quantification of AT8- and AT100-positive area fractions in the indicated brain regions. Levels of hyperphosphorylated tau significantly increased with age, with a dramatic rise observed in 10-month-old hSyn mice compared to age-matched WT controls (see Supplementary **Fig. S2**). Bar graphs represent mean ± SEM. Statistical analysis was performed using two-way ANOVA with Tukey’s post-hoc test (*p < 0.05, **p < 0.01, ***p < 0.001 for age-dependent comparisons within the same group; ^#^p < 0.05, ^##^p < 0.01, ^###^p < 0.001, ^####^p < 0.0001 for comparisons between hSyn and WT littermates).

Tau hyperphosphorylation disrupts microtubule stability, promoting abnormal aggregation of tau into paired helical filaments (PHFs) that ultimately form neurofibrillary tangles (NFTs) [54]. To assess the development of tau pathology in hSyn mice, we performed immunofluorescence (IF) staining for hyperphosphorylated tau using AT8 and AT100 antibodies. The AT8 antibody specifically recognizes tau phosphorylated at Ser202 and Thr205, detecting both soluble hyperphosphorylated tau and PHF-tau [55], whereas the AT100 antibody detects tau phosphorylation at Thr212 and Ser214, marking more advanced stages of tau pathology and selectively identifying later-stage tau aggregates, including mature NFTs [56]. Hyperphosphorylated tau was undetectable up to 5 months but progressively increased thereafter, with significant accumulation in the cortex, hippocampus, and midbrain of hSyn mice (Fig. 1). By 10 months, robust tau pathology emerged, characterized by cytoplasmic AT8- and AT100-positive inclusions with morphology resembling NFTs (Fig. 1A, B). Quantitative analysis revealed a significant increase in AT8 and AT100 immunoreactivity in hSyn mice compared with age-matched WT controls (Fig. 1C-H). In contrast, WT littermates showed no detectable tau pathology even at 20 months of age (Supplementary **Fig. S2**). Together, these findings demonstrate a progressive, age-dependent development of tau pathology in the hSyn mice.

**Figure 2:**
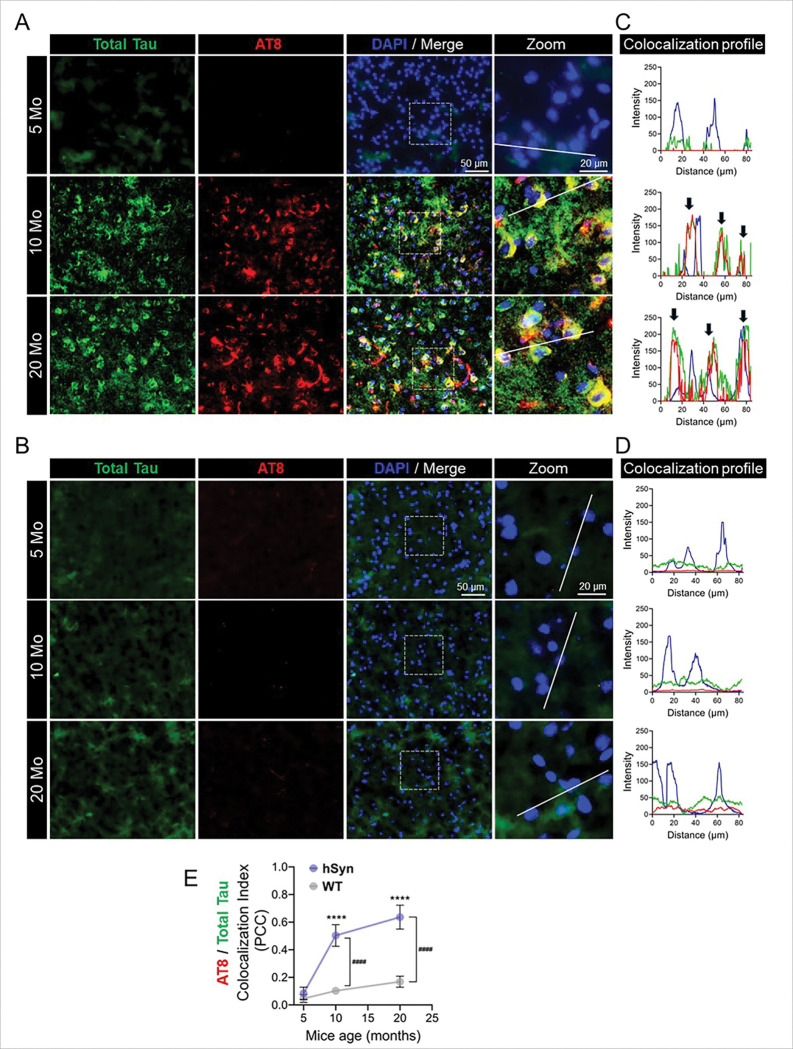
Colocalization of hyperphosphorylated tau with total tau in hSyn mice. (**A**, **B**) Representative co-immunofluorescence images of hSyn and WT mice cortex co-stained for hyperphosphorylated tau (AT8, red) and total tau (chicken-polyclonal tau, green), with DAPI staining for nuclei (blue). Scale bars: 50 μm; 20 μm for inset images. (**C**, **D**) Intensity profile plots of colocalization of AT8, total tau, and DAPI along an 82 μm segment (indicated by white lines), analyzed using ImageJ software. Significant colocalization of hyperphosphorylated tau (AT8, red) with chicken polyclonal tau (green) is evident in 10- and 20-month-old hSyn mice (black arrows), whereas younger (5-month-old) mice and WT mice at all ages show minimal colocalization and lack substantial hyperphosphorylated tau. (**E**) Quantitative analysis of AT8 and total tau colocalization using Pearson’s correlation coefficient (PCC). PCC values are presented as mean ± SD, based on six regions of interest in the cortex. Statistical analysis was performed using two-way ANOVA with Tukey’s post-hoc test (****p < 0.0001 for age-dependent comparisons within the same group; ^####^p < 0.0001 for comparisons between hSyn and WT littermates).

To validate the specificity of AT8 and AT100 immunoreactivity, brain sections from hSyn mice were co-stained with AT8 or AT100 and a total tau antibody (chicken polyclonal tau) (Fig. 2; & Supplementary **Fig. S3**). Colocalization analyses revealed strong spatial overlap between total tau and hyperphosphorylated tau (AT8 and AT100) in 10- and 20-month-old hSyn mice, as shown by colocalization plot profiles (Fig. 2C, E; Supplementary **Fig. S3C)**. In contrast, negligible colocalization was observed at 5 months, indicating the absence of detectable hyperphosphorylated tau at early stages. WT mice showed no significant colocalization at any age (Fig. 2B, D, E; Supplementary **Fig. S3B, D**).

### Immunohistochemical evidence of progressive tau accumulation

**Figure 3:**
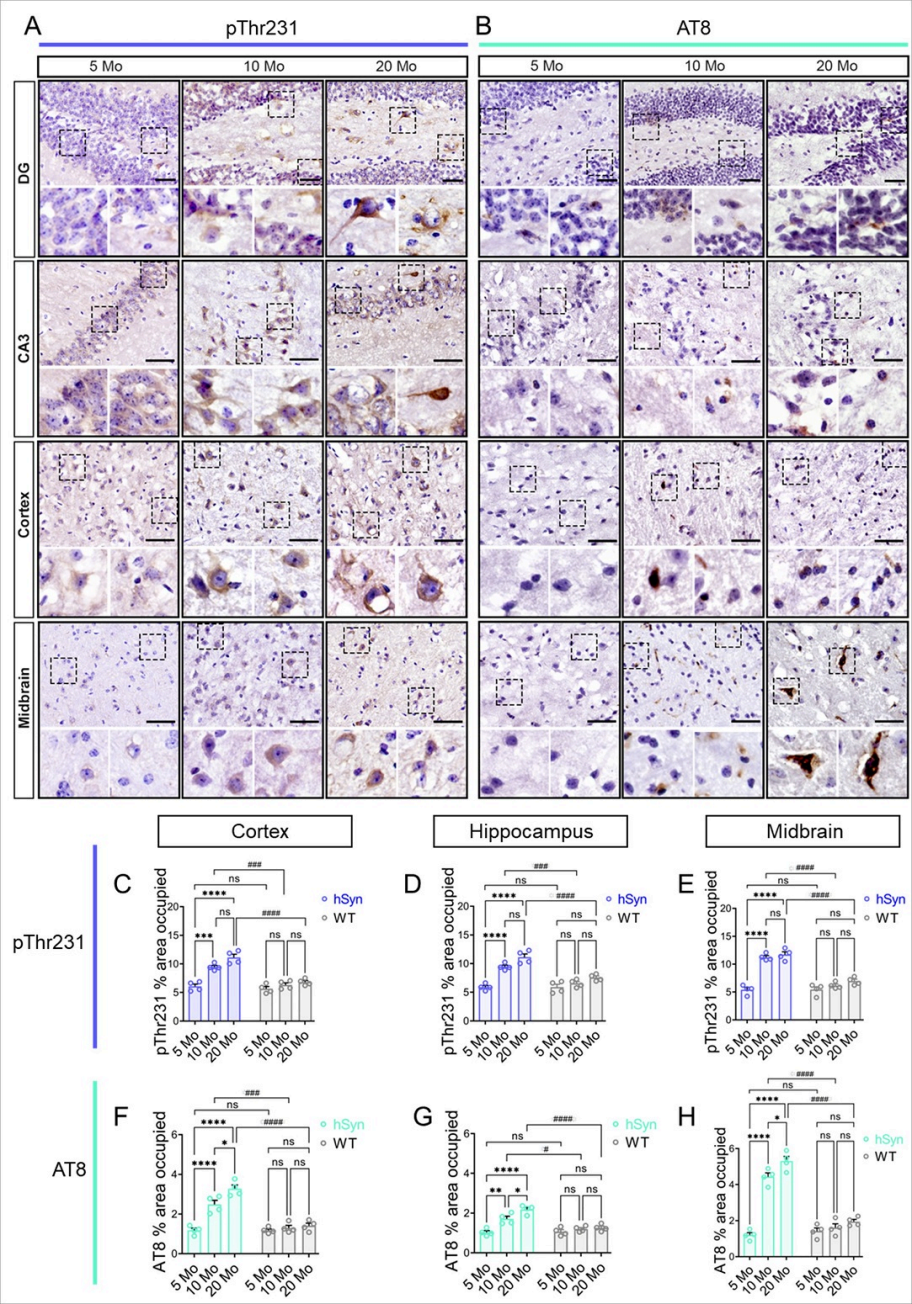
Immunohistochemical evidence of progressive accumulation of hyperphosphorylated tau inclusions in hSyn mice. (**A**, **B**) Representative immunohistochemistry images showing age-dependent accumulation and maturation of tau pathology, detected by pThr231 and AT8 staining in the cortex, hippocampus, and midbrain of hSyn mice at 5, 10, and 20 months. (**C**-**H**) Quantitative analysis of pThr231 and AT8 immunoreactivity demonstrates a significant, age-dependent increase in tau phosphorylation across these brain regions. Hyperphosphorylated tau levels were significantly elevated at 10 and 20 months compared to younger (5-month-old) hSyn mice and age-matched WT controls (see Supplementary **Fig. S4** & **S5**). Scale bar = 50 μm. Results are presented as mean ± SEM. Statistical analyses were performed using Two-way ANOVA with Tukey’s post-hoc test (*p < 0.05, **p < 0.01, ***p < 0.001 for age-dependent comparisons within the same group; (^#^p < 0.05, ^##^p < 0.01, ^###^p < 0.001, ^####^p < 0.0001 for comparisons between hSyn and WT littermates).

The temporal progression of tau pathology was further evaluated by immunohistochemistry (IHC) using phospho-Thr231 (pThr231) and AT8 antibodies. In hSyn mice, pThr231 immunoreactivity was detectable as early as 5 months, whereas AT8-positive hyperphosphorylated tau was not evident until 10 months (Fig. 3). In contrast, WT littermates showed no detectable hyperphosphorylated tau even at 20 months (Supplementary **Fig. S4, S5**).

Tau hyperphosphorylation promotes tau aggregation and the subsequent formation of NFTs, which evolve through three maturation stages: pre-tangles, characterized by diffuse cytoplasmic tau staining in morphologically normal neurons; mature tangles, which occupy the entire cytoplasm and assume the shape of the neuron; and ghost tangles, composed of tau fibrils left behind after neuronal death [54, 57]. At early stages, pThr231 and AT8 staining in hSyn mouse brains exhibited diffuse, cytoplasmic tau localization, likely corresponding to pre-tangles. By 20 months, dense inclusions occupying the entire cytoplasm were evident in the midbrain, cortex, and hippocampus, indicative of mature NFTs (Fig. 3A, B). Quantitative analysis of IHC staining corroborated the immunofluorescence data, revealing a progressive, age-dependent increase of tau pathology in hSyn mice (Fig. 3C-H), while WT mice showed no significant hyperphosphorylated tau across all ages (Supplementary **Fig. S4**, **S5**).

### Biochemical evidence of progressive tau pathology in hSyn mice

**Figure 4:**
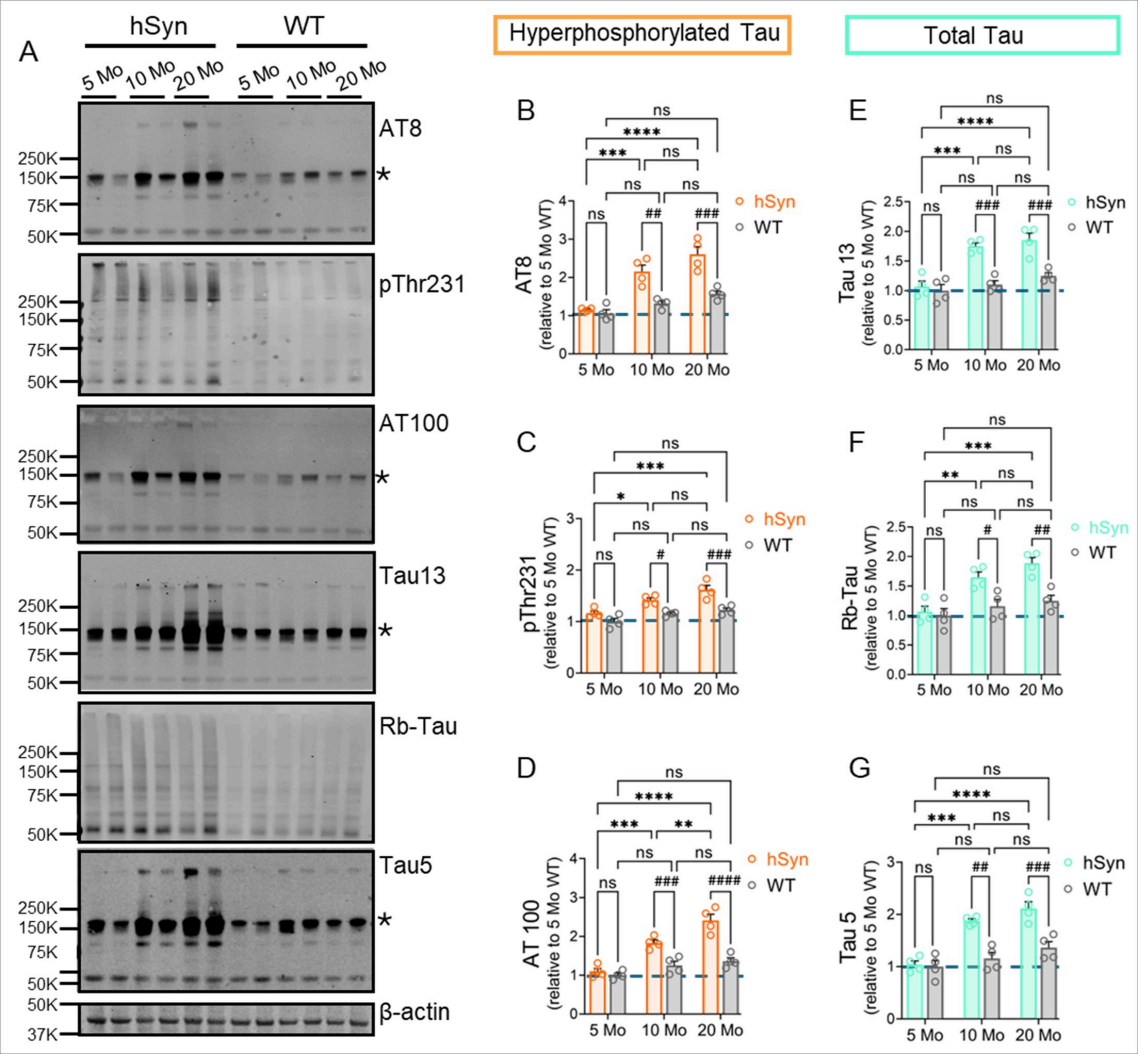
Western blot evidence of age-dependent pathological tau accumulation in hSyn mice. (**A**) Representative Western blot of PBS-soluble brain homogenates from 5-, 10-, and 20-month-old hSyn mice and WT littermates probed with hyperphosphorylated tau (AT8, pThr231, and AT100) and total tau (Tau13, rabbit-polyclonal tau, and Tau5) antibodies. (**B-D**) Quantification of hyperphosphorylated tau demonstrates significantly elevated AT8, pThr231, and AT100 signals in 10- and 20-month-old hSyn mice compared with 5-month-old hSyn and age-matched WT controls. (**E-G**) Quantification of total tau levels using the Tau13, Rb-Tau, and Tau5 antibodies shows increased tau levels in 10- and 20-month-old hSyn mice compared to the 5-month-old hSyn group and age-matched WT animals. Asterisks indicate tau protein bands that may overlap with mouse IgG. Bar graphs represent mean ± SEM (*n* = 4 per group). Statistical analyses were performed using two-way ANOVA with Tukey’s post-hoc test (*p < 0.05, **p < 0.01, ***p < 0.001, ****p < 0.0001 for age-dependent comparisons within the same group; ^#^p < 0.05, ^##^p < 0.01, ^###^p < 0.001, ^####^p < 0.0001 for comparisons between hSyn and WT littermates).

To complement the histological findings, we performed western blot analysis on PBS-soluble fractions of whole-brain homogenates from hSyn mice, utilizing a range of commercially available classical tau markers (Fig. 4). Hyperphosphorylated tau levels, assessed using AT8, pThr231, and AT100 antibodies, showed a clear age-dependent increase in hSyn mice consistent with the IF and IHC results (Fig. 4B-D). AT8 and pThr231 immunoreactivity increased significantly between 5 and 10 months, with no further significant increase between 10 and 20 months. Low levels of AT8-positive tau were detectable at 5 months, followed by a robust elevation at 10 months (p < 0.001) (Fig. 4B). pThr231 tau showed a moderate but significant rise over the same interval (p < 0.05) (Fig. 4C). AT100 immunoreactivity increased significantly at all ages, with a marked surge between 5 and 10 months (p < 0.001), and a further significant increase between 10 and 20 months (p < 0.01) (Fig. 4D). In contrast, WT littermates exhibited no elevated levels of hyperphosphorylated tau even at 20 months (Fig. 4B-D).

Total tau levels were assessed using Tau13, rabbit polyclonal tau (Rb-Tau), and Tau5 antibodies. Total tau levels increased significantly with age in hSyn mice, showing a marked increase between 5 and 10 months (Fig. 4E-G). At 10 and 20 months, total tau levels were significantly higher in hSyn mice than in age-matched WT controls, whereas no significant differences were noted at 5 months. Collectively, these biochemical results are consistent with IF and IHC analyses, confirming a progressive, age-dependent accumulation of pathological tau in hSyn mice.

Given the progressive increase in total and hyperphosphorylated tau, we next focused on whether these age-dependent changes were accompanied by the accumulation of insoluble tau and α-Syn aggregates.

### Aged hSyn mice accumulate insoluble tau and α-Syn aggregates

**Figure 5:**
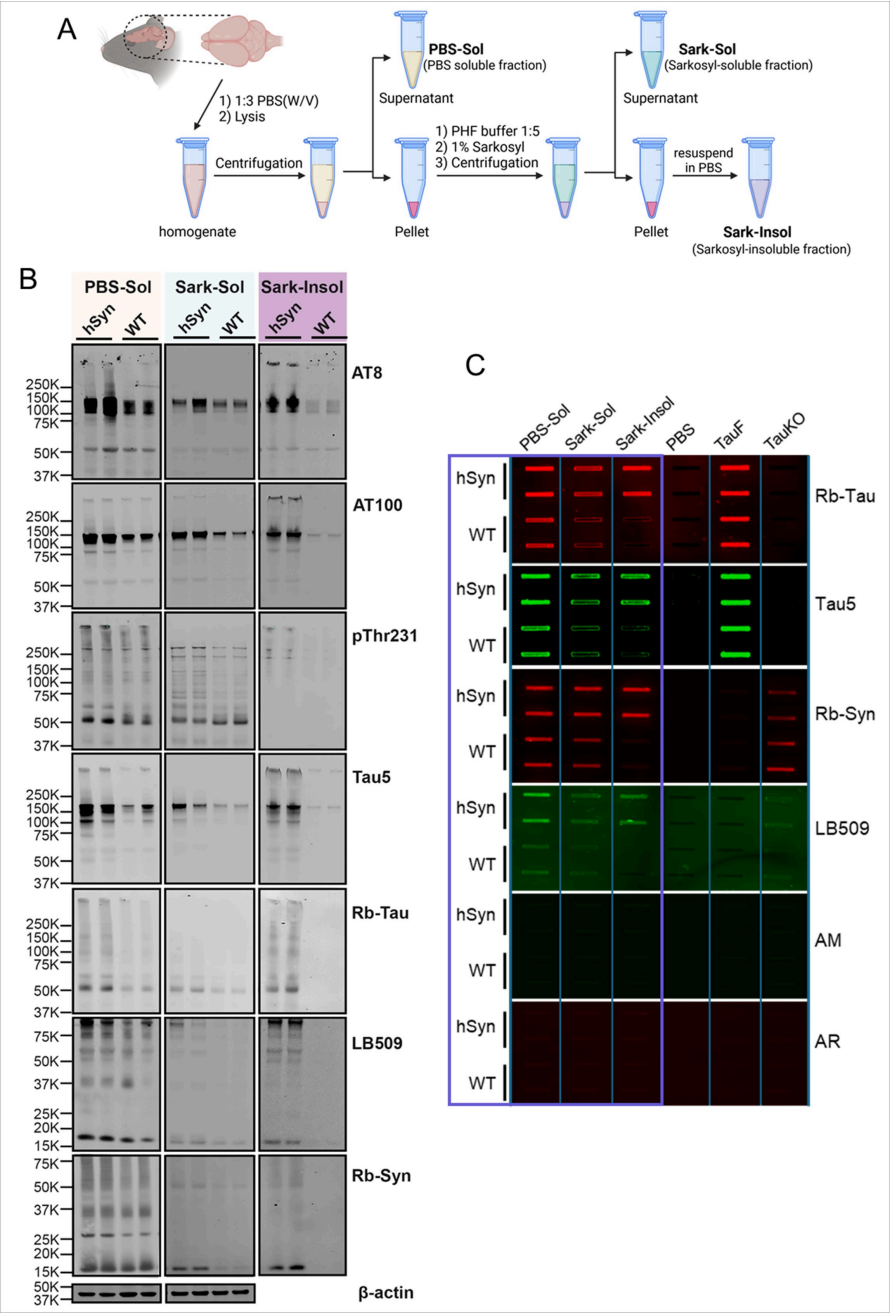
Accumulation of insoluble tau and α-Syn aggregates in the brains of hSyn mice. (A) Schematic illustration of the sequential extraction method used to isolate PBS-soluble (PBS-Sol), sarkosyl-soluble (Sark-Sol), and sarkosyl-insoluble (Sark-Insol) protein fractions from mouse brain tissue. (B) Representative Western blot images of protein fractions from 20-month-old hSyn and WT mice. Blots were probed with antibodies against hyperphosphorylated tau (AT8, AT100, pThr231), total tau (Tau5, rabbit polyclonal tau), and α-Syn (LB509 and rabbit polyclonal α-Syn). hSyn mice exhibited marked accumulation of pathological tau and α-Syn in both Sark-Sol and Sark-Insol fractions, whereas WT littermates showed no detectable tau and α-Syn in the Sark-Insol fraction. (C) Filter trap assay of PBS-Sol, Sark-Sol, and Sark-Insol protein fractions from 20-month-old hSyn and WT mice, immunoblotted using antibodies against total tau (rabbit polyclonal abbreviated as Rb-Tau, Tau5) and α-Syn (rabbit polyclonal α-Syn and LB509). AM and AR represent anti-mouse and anti-rabbit secondary antibodies, respectively.

To assess the biochemical properties of insoluble aggregates, we focused on 20-month-old hSyn mice, given that tau pathology peaks at this age. Sequential extraction of brain homogenates was performed to obtain PBS-soluble (PBS-Sol), sarkosyl-soluble (Sark-Sol), and sarkosyl-insoluble (Sark-Insol) fractions (Fig. 5A). Homogenates were first centrifuged to separate larger tau aggregates into the pellet, while the PBS-Sol species, including monomeric, oligomeric, and HMW proteins, remained in the supernatant. The pellet was then treated with sarkosyl detergent and subjected to high-speed centrifugation to isolate the Sark-Insol fraction.

Samples were analyzed by immunoblotting using phosphorylation-dependent and total tau antibodies (AT8, AT100, pThr231, Tau5, and Rb-Tau) as well as α-Syn antibodies (LB509 and Rb-Syn). Western blot analysis revealed a marked accumulation of hyperphosphorylated and total tau, as well as α-synuclein, in both the Sark-Sol and Sark-Insol fractions of hSyn mice, while WT littermates lacked detectable insoluble tau or α-Syn (Fig. 5B). Total tau (Tau5, Rb-Tau) was present across all fractions but showed a shift toward higher-molecular-weight, insoluble species in hSyn mice. α-Syn (LB509, Rb-Syn) was also highly enriched in hSyn mice compared to the WT littermates, with increased levels in the Sark-Insol fraction. These results demonstrate the accumulation of insoluble tau and α-Syn aggregates in the brains of aged hSyn mice.

To complement the accumulation of Sark-Insol aggregates, we performed a filter trap assay on sequentially extracted fractions using total tau (Rb-Tau, Tau5) and α-Syn (LB509, Rb-Syn) antibodies from 20-month-old hSyn and WT mouse brains (Fig. 5C). Strong retention of tau and α-Syn signals was detected in the Sark-Sol and Sark-Insol fractions of hSyn mice, while WT and Tau knockout (TauKO) controls showed no detectable signals. Together, these results confirm that aged hSyn mice accumulate insoluble tau and α-syn species.

### Aged hSyn mice accumulate insoluble fibrillar aggregates composed of α-Syn and tau

**Figure 6:**
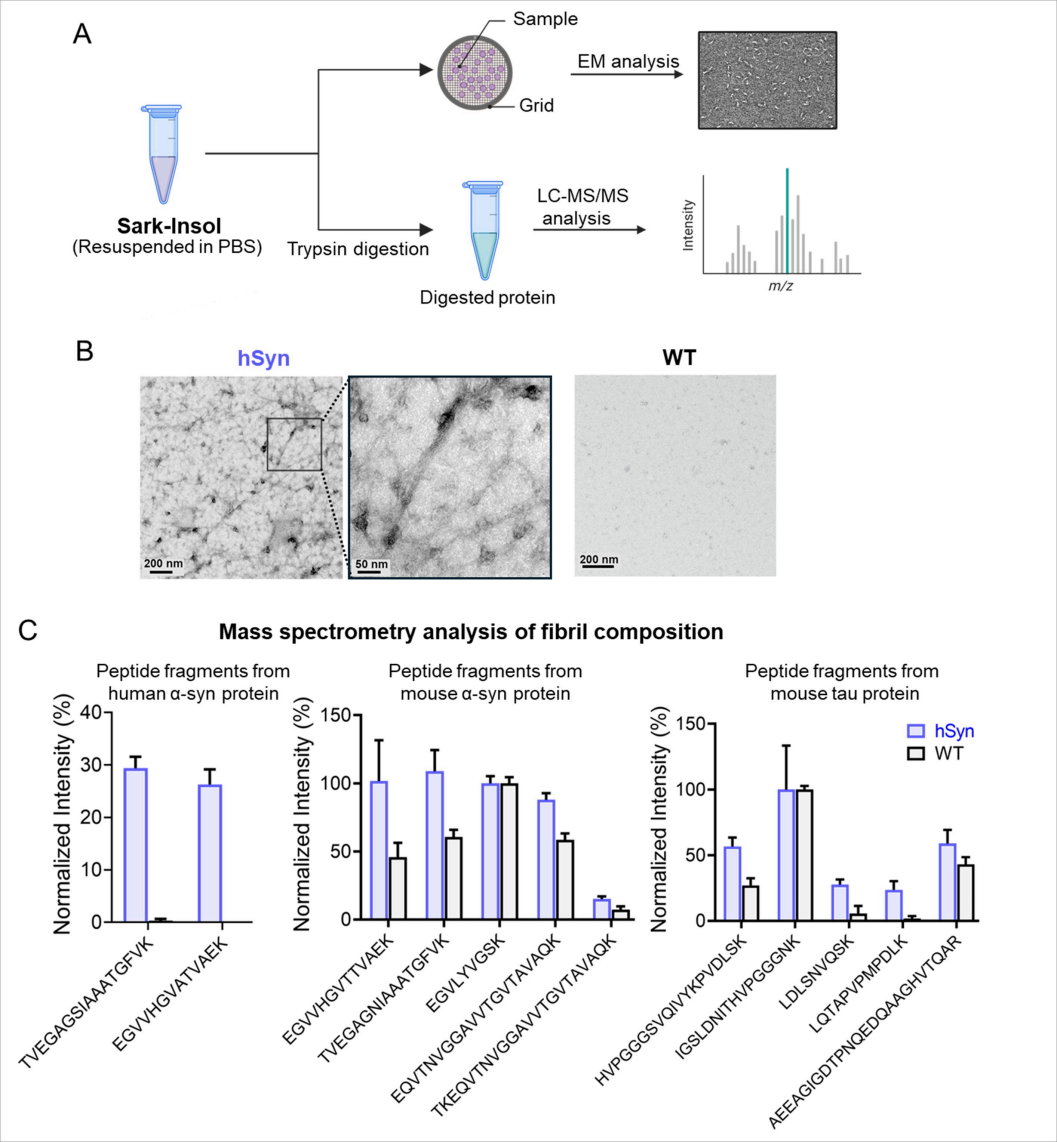
Morphological and molecular characterization of sarkosyl-insoluble aggregates in hSyn mouse brains. (**A**) Schematic diagram outlining the workflow for analyzing the sarkosyl-insoluble (Sark-Insol) fraction using electron microscopy (EM) for morphological assessment and LC-MS/MS for molecular composition analysis. (**B**) Representative EM images showing fibrillar structures in the Sark-Insol fraction of 20-month-old hSyn mouse brains, visualized using uranyl acetate negative staining. (**C**) LC-MS/MS analysis of trypsin-digested Sark-Insol fractions from 20-month-old hSyn and WT mouse brains. Samples were digested with trypsin for 3 hours under native conditions, and the resulting peptides were subjected to mass spectrometry. Intensity of each α-Syn peptide was normalized with the proteotypic peptide EGVLYBGSK, while IGSLDNITHVPGGGNK was used for tau. The results demonstrate co-aggregation of α-Syn and tau within the insoluble protein aggregates.

To further assess the structural and molecular composition of insoluble aggregates in aged hSyn mice, we analyzed the Sark-Insol fractions using transmission electron microscopy (EM) and liquid chromatography–tandem mass spectrometry (LC–MS/MS) (Fig. 6A). EM imaging revealed abundant fibrillar structures in hSyn mice brains (Fig. 6B). To determine the molecular composition of these fibrils, Sark-Insol fractions were subjected to trypsin digestion followed by LC–MS/MS analysis (Fig. 6C). Peptide mapping revealed enrichment of both α-Syn and tau peptides in the insoluble fraction of hSyn mice. The relative abundance of α-Syn and tau peptides, normalized to their respective proteotypic reference peptides (EGVLYVGSK for α-Syn [50] and IGSLDNITHVPGGGNK for tau [51]), confirmed the co-presence of α-Syn and tau in the Sarkosyl-Insol protein pool. Collectively, these results demonstrate that aged hSyn mouse brains accumulate insoluble fibrillar aggregates enriched in both α-Syn and tau.

## Discussion

Although tau pathology is increasingly recognized in synucleinopathies, its age-dependent progression, particularly in models expressing WT α-Syn, remains poorly understood. Most prior studies have relied on mutant α-Syn mouse models that exhibit aggressive phenotypes but fail to recapitulate the gradual progression of human synucleinopathies. The hSyn mouse, which overexpresses human WT α-Syn under the neuron-specific Thy-1 promoter, develops progressive neuropathological and behavioral deficits closely resembling synucleinopathies. α-Syn pathology emerges by 6 months in key brain regions, including cortex, hippocampus, and substantia nigra [31], with microglial activation evident in the striatum by 10 months [6]. By 12 months, reductions in dopamine and serotonin levels are observed [34], accompanied by declines in locomotor activity, motor coordination, and olfactory performance by 8 months [4, 23, 31, 34].

Our lab and others have demonstrated the co-occurrence of α-Syn and tau aggregates in postmortem brain tissues from patients with synucleinopathies [14, 58–61]. Preclinical studies further support the role of tau as an active contributor to disease progression in α-Syn–driven disorders [21, 30, 53, 62, 63]. The presence of tau as a co-pathology has been associated with more severe symptoms, accelerated neurodegeneration, and reduced responsiveness to α-Syn directed therapies [14, 18–20]. However, the mechanistic and temporal relationship between α-Syn and tau aggregation remains incompletely understood.

In the present study, we systematically characterized the temporal evolution of tau pathology in the hSyn mouse model to determine how α-Syn overexpression influences tau aggregation. Consistent with earlier reports, our data showed that α-Syn levels remain stable until 10 months but increase significantly by 20 months, suggesting an age-dependent progression of α-Syn pathology. Immunofluorescence analyses revealed negligible tau phosphorylation at 5 months, followed by marked accumulation of AT8- and AT100-positive tau in the cortex, hippocampus, and midbrain by 10 months. Co-localization of hyperphosphorylated tau with total tau at 10 and 20 months confirmed progressive tau aggregation, while WT littermates exhibited no detectable tau pathology even at 20 months.

Immunohistochemical and biochemical analyses further revealed distinct stages of tau pathology that parallel those seen in human tauopathies. Early diffuse cytoplasmic staining with pThr231 and AT8 antibodies suggested pre-tangle formation, while dense, compact inclusions consistent with mature NFTs appeared by 20 months. This temporal evolution from soluble to fibrillar tau species underscores a pathological process driven by sustained α-Syn accumulation. Importantly, the absence of tau pathology in WT littermates confirms that tau aggregation arises as a downstream effect of α-Syn overexpression and aggregation, rather than normal aging.

Biochemical fractionation and structural analyses provided further insight into the composition and maturation of these aggregates. Western blotting and filter trap assays demonstrated that both α-Syn and tau became increasingly insoluble with age, accumulating in Sark-Insol fractions. Electron microscopy revealed abundant fibrillar structures in brains of aged hSyn mice, and LC–MS/MS confirmed the presence of both α-Syn and tau in the insoluble fraction. These findings provide biochemical evidence for co-occurrence of α-Syn and tau within pathological protein aggregates in aged hSyn mice, consistent with observations in synucleinopathies [14].

Collectively, these results have important implications for understanding the overlapping mechanisms underlying PD, DLB, and related disorders. The hSyn mouse model naturally develops both α-Syn and tau pathologies in an age-dependent manner, without requiring exogenous seeding, making it a valuable platform for studying disease progression and evaluating therapeutic interventions. Previous studies have shown that tau is essential for the progressive synaptic and memory deficits in transgenic models of α-Syn pathology [30]. Given the central role of tau in mediating α-Syn–driven toxicity, therapies targeting only α-Syn may be insufficient. Our findings reinforce the emerging concept that tau pathology is a common and functionally significant feature across synucleinopathies and suggest that therapeutic strategies targeting tau or simultaneously modulating both α-Syn and tau aggregation pathways, may more effectively mitigate neurodegeneration in synucleinopathies.

In summary, we demonstrate that α-Syn overexpression in hSyn mice triggers a cascade of tau hyperphosphorylation, aggregation, and NFT formation, ultimately leading to the co-assembly of α-Syn and tau into insoluble fibrillar aggregates. These results provide direct experimental evidence that tau acts as a critical mediator of α-Syn–driven neurodegeneration. The hSyn mouse model thus represents a physiologically relevant platform for exploring α-Syn–tau crosstalk and for developing tau-targeted or dual-pathway therapeutic strategies for synucleinopathies and related disorders. Ongoing studies in our laboratory aim to elucidate the molecular mechanisms through which α-Syn overexpression promotes tau pathology and to investigate the co-aggregation of α-Syn and tau aggregates within both soluble and insoluble fractions, thereby advancing our understanding of disease mechanisms and guiding future therapeutic development.

## Conclusion

This study demonstrates that overexpression of human WT α-Syn in the hSyn mouse model induces a progressive cascade of tau hyperphosphorylation, aggregation, and neurofibrillary tangle formation. Through complementary histopathological, biochemical, and structural analyses, we delineate a temporal sequence from early soluble tau species to mature fibrillar inclusions. The absence of comparable pathology in wild-type littermates underscores that tau aggregation is a downstream consequence of human α-Syn overexpression rather than normal aging. Together, these findings establish a mechanistic link between α-Syn and tau aggregation, identifying tau as an active mediator of α-Syn–driven neurodegeneration. By developing both α-Syn and tau pathologies without exogenous seeding, the hSyn model represents a relevant model for investigating the molecular interplay between α-Syn and tau in PD and related synucleinopathies. Targeting tau pathology, alone or in combination with α-Syn, may therefore represent a promising therapeutic approach to slow or halt neurodegeneration in PD, DLB, and related disorders.

## Supplementary Material

Supplementary Files

This is a list of supplementary files associated with this preprint. Click to download.

• SupplementaryInformation.docx

• SupplementaryfileFulluncroppedGelsandBlotsimage.docx

## Data Availability

All data associated with this study are present in the paper or the Supplementary Information.

## Abbreviations

α-Syn: Alpha-synuclein
ACN: Acetonitrile
AD: Alzheimer’s disease
AGC: Automatic gain control
ANOVA: Analysis of variance
ARC: Animal Resources Center
AT8: Antibody recognizing tau phosphorylated at Ser202/Thr205
AT100: Antibody recognizing tau phosphorylated at Thr212/Ser214
BSA: Bovine serum albumin
CBD: Corticobasal degeneration
CBF: Cerebral blood flow
CDK5: Cyclin-dependent kinase 5
CNS: Central nervous system
DAB: 3,3’-Diaminobenzidine
DAPI: 4′,6-diamidino-2-phenylindole
DLB: Dementia with Lewy bodies
DMSO: Dimethyl sulfoxide
EDTA: Ethylenediaminetetraacetic acid
EM: Electron microscopy
FA: Formic acid
FTD: Frontotemporal dementia
GSK3β: Glycogen synthase kinase 3 beta
HCD: Higher-energy collisional dissociation
HMW: High-molecular-weight
hSyn: mThy1-hSNCA transgenic mouse expressing human WT α-Syn (C57BL/6N-Tg (Thy-1-SNCA)15Mjff/J)
IACUC: Institutional Animal Care and Use Committee
IHC: Immunohistochemistry
IF: Immunofluorescence
IT: Ion time / injection time
JAX: Jackson Laboratory
LC–MS/MS: Liquid chromatography–tandem mass spectrometry
LFQ: Label-free quantification
MoM: Mouse-on-mouse blocking reagent
MS: Mass spectrometry
NFTs: Neurofibrillary tangles
NIH: National Institutes of Health
NCE: Normalized collision energy
OCT: Optimal cutting temperature compound
PBS: Phosphate-buffered saline
PBS-Sol: PBS-soluble fraction
PBST / TBS-T: PBS or TBS with Tween-20
PD: Parkinson’s disease
PHF: Paired helical filaments
pThr231: Tau phosphorylated at threonine 231
PTMs: Post-translational modifications
RT: Room temperature
SD / SEM: Standard deviation / Standard error of the mean
Sark-Sol: Sarkosyl-soluble fraction
Sark-Insol: Sarkosyl-insoluble fraction
SNCA: Human alpha-synuclein gene
TauKO: Tau knockout
TBS: Tris-buffered saline
TEM: Transmission electron microscopy
UHPLC: Ultra-high-performance liquid chromatography
UTMB: University of Texas Medical Branch
WT: Wild-type
